# Escores de Risco Cardiovascular entre Adultos Assintomáticos com Hemofilia

**DOI:** 10.36660/abc.20230004

**Published:** 2023-09-04

**Authors:** Ricardo Mesquita Camelo, Camila Caram-Deelder, Bruna Pontes Duarte, Marilia Carolina Braga de Moura, Neuza Cavalcanti de Moraes Costa, Iris Maciel Costa, Ana Maria Vanderlei, Tania Maria Rocha Guimarães, Samantha Gouw, Suely Meireles Rezende, Johanna van der Bom

**Affiliations:** 1 Faculdade de Medicina Universidade Federal de Minas Gerais Belo Horizonte MG Brasil Faculdade de Medicina, Universidade Federal de Minas Gerais, Belo Horizonte, MG – Brasil; 2 HEMOPE Recife PE Brasil Fundação de Hematologia e Hemoterapia de Pernambuco (HEMOPE), Recife, PE – Brasil; 3 Department of Clinical Epidemiology Leiden University Medical Center Leiden Países Baixos Department of Clinical Epidemiology, Leiden University Medical Center, Leiden – Países Baixos; 4 Sanquin LUMC Leiden Países Baixos Jon J van Rood Center for Clinical Transfusion Research, Sanquin/LUMC, Leiden – Países Baixos; 5 Faculdade de Enfermagem Nossa Senhora das Graças Universidade de Pernambuco Recife PE Brasil Faculdade de Enfermagem Nossa Senhora das Graças, Universidade de Pernambuco, Recife, PE – Brasil; 6 Department of Pediatric Hematology Emma Children’s Hospital University of Amsterdam Amsterdã Países Baixos Department of Pediatric Hematology, Emma Children’s Hospital, Amsterdam UMC, University of Amsterdam,Amsterdã – Países Baixos

**Keywords:** Hemofilia A, Hemofilia B, Prevenção Primária, Fatores de Risco de Doenças Cardíacas

## Abstract

**Fundamento:**

A taxa de mortalidade de pessoas com hemofilia (PCH) no Brasil está diminuindo, mas a incidência relativa de mortes associadas a doenças cardiovasculares (DCV) tem aumentado.

**Objetivos:**

Nosso objetivo foi descrever o escore de risco de DCV de PCHs de acordo com a ferramenta *Pooled Cohort Equations Risk* (PCER) *Calculator* e suas recomendações de tratamento. Além disso, foram comparadas as estimativas da PCER com o respectivo escore de risco de Framingham (FRS).

**Métodos:**

Este estudo transversal incluiu PCHs do sexo masculino, com idade igual ou superior a 40 anos, tratados no Centro de Tratamento Integral de Hemofilia de Pernambuco (Recife/Brasil). PCHs com um evento cardiovascular prévio ou colesterol lipídico de baixa densidade ≥ 5,0 mmol/L foram excluídas. Entrevistas, revisões de prontuários médicos e exames de sangue foram realizados. A ferramenta PCER foi utilizada para estimar o risco de DCV e compará-lo com o respectivo FRS. Um valor de p < 0,05 foi aceito como estatisticamente significativo.

**Resultados:**

Trinta PCHs foram incluídas. A idade mediana foi de 51,5 [intervalo interquartil-IIQ; 46,0-59,5] anos. A prevalência de obesidade, hipertensão arterial sistêmica, diabetes mellitus, hipertrigliceridemia, hipercolesterolemia e hipoHDLemia foi de 20%, 67%, 24%, 14%, 47% e 23%, respectivamente. O escore mediano da PCER foi de 6,9% [IIQ; 3,1-13,2], com 50% de alto risco (PCER ≥ 7,5%). O uso de estatina foi sugerido para 54% das PCHs. A pressão arterial estava mal controlada em 47% das PCHs. A concordância entre PCER e FRS foi de 80% (κ = 0,60; p = 0,001).

**Conclusões:**

Metade dos homens com hemofilia, com 40 anos de idade ou mais, teve um alto risco de desenvolver DCV em 10 anos, com fortes recomendações para melhorar o controle da dislipidemia e da pressão arterial.

**Figura Central:**
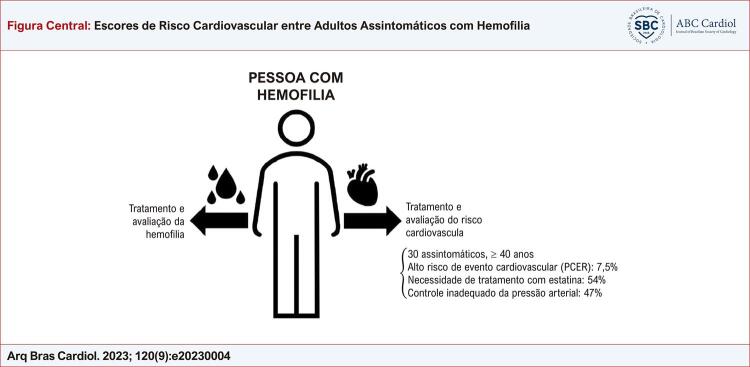
: Escores de Risco Cardiovascular entre Adultos Assintomáticos com Hemofilia

Desde o desenvolvimento de produtos mais eficazes e seguros para tratar o tratamento da hemofilia, as pessoas que vivem com a doença estão vivendo mais. Além disso, esses indivíduos passaram a desenvolver doenças cardiovasculares características do envelhecimento. Propomos que hematologistas e outros profissionais de saúde que cuidam de indivíduos com hemofilia realizem avaliações regulares e formais dos fatores de risco cardiovascular nesses pacientes e, quando presentes, recomendamos que tais condições sejam tratadas de acordo com protocolos estabelecidos. Quando necessário, os pacientes devem ser encaminhados para um especialista. (PCER, Pooled Cohort Equations Risk)

## Introdução

A hemofilia é um distúrbio hemorrágico raro hereditário ligado ao cromossomo X, caracterizado pela redução ou ausência da atividade do fator de coagulação VIII (na hemofilia A) ou do fator IX (na hemofilia B).^1^ Em 2020, havia 209.614 pessoas com hemofilia (PCH) em todo o mundo, das quais 165.379 tinham hemofilia A, 33.076 tinham hemofilia B e os 11.159 restantes tinham um tipo desconhecido de hemofilia.^2^ A apresentação clínica mais comum da doença é a hemorragia espontânea, principalmente nas articulações, mas também em outros locais (por exemplo, epistaxe ou sistêmica).^1^ Portanto, a hemofilia é considerada uma doença potencialmente grave por causa de suas morbidades e mortalidade.

A terapia de reposição de fator proporcionou um aumento na expectativa de vida das PCHs.^3-5^ Sendo assim, PCHs estão vivendo mais e a incidência de eventos cardiovasculares (por exemplo, infarto do miocárdio e acidente vascular cerebral isquêmico) tem aumentado.^3-5^De acordo com diretrizes internacionais, doenças cardiovasculares (DCV) devem ser tratadas com medicamentos antitrombóticos durante o evento agudo e para prevenção secundária.^6,7^ No entanto, não existem estudos controlados randomizados sobre o tratamento ideal das DCVs entre PCHs. Os médicos podem basear seus tratamentos em opiniões de especialistas, equilibrando os riscos de eventos hemorrágicos (evitando ou reduzindo a profilaxia de reposição de fator e/ou prescrevendo medicamentos antitrombóticos) e coagulação (prescrevendo profilaxia de reposição de fator e/ou evitando medicamentos antitrombóticos).^8,9^ Identificar e tratar fatores de risco de DCVs para preveni-las pode representar um desafio menor do que tratar um evento de DCV já instalado, uma vez que o controle de peso, a cessação do tabagismo, tratamentos de hipertensão arterial sistêmica (HAS), diabetes mellitus (DM) e dislipidemia como prevenção primária não estão associados ao risco aumentado de hemorragias.

O Brasil tem uma das maiores populações hemofílicas do mundo (n = 13.149).^2^ Na última década, após a adoção das recomendações de atendimento para o tratamento da hemofilia, a expectativa de vida da PCH brasileira aumentou.^5^ Como consequência, a mortalidade relacionada a DCVs também tem aumentado proporcionalmente.^5^ O objetivo da análise atual do Estudo HemoCardio foi descrever o escore de risco para DCV entre PCHs utilizando a ferramenta *Pooled Cohort Equations Risk* (PCER) *Calculator* e suas recomendações de tratamento. Uma análise secundária comparou esses resultados com o escore de risco de Framingham (FRS).

## Métodos

### Desenho do estudo, cenário e elegibilidade do paciente

O Estudo HemoCardio transversal foi realizado no Centro de Tratamento Integral de Hemofilia de Pernambuco (CTIH-HEMOPE), em Recife/Brasil. Em 2016, 711 PCHs estavam registradas no estado Pernambuco, 227 das quais tinham 40 anos ou mais, e aproximadamente 76 foram acompanhadas no CTIH-HEMOPE.^10^ O estudo foi oferecido a todos os homens com hemofilia com idade igual ou superior a 30 anos, registrados no ambulatório durante consulta eletiva no CTIH-HEMOPE entre 1º de agosto de 2018 e 31 de julho de 2019, totalizando 82 participantes. Na análise atual, foram usados dados de homens com hemofilia com 40 anos ou mais, uma vez que representam a faixa etária-alvo para a avaliação do PCER (Figura Central). Pacientes com histórico de DCV ou colesterol lipídico de baixa densidade (LDLc) de 5,0 mmol/L ou mais alto foram excluídos, pois essas características indicam muito alto risco de evento de DCV precocemente, e o cálculo do PCER não é recomendado para esses casos.^11,12^ Todos os dados foram coletados por meio de um formulário padronizado.

### Dados relacionados à hemofilia

Uma descrição detalhada dos dados relacionados à hemofilia pode ser encontrada no Material Suplementar.

### Perfil de fator de risco cardiovascular

Uma descrição detalhada do perfil do fator de risco cardiovascular pode ser encontrada no Material Suplementar.

### Ferramentas de estimativa de risco cardiovascular

A ferramenta PCER Calculator (www.cvriskcalculator.com) foi usada para estimar o risco de DCV em dez anos (doença cardíaca ou acidente vascular encefálico), pressupondo-se que a pessoa não teve um ataque cardíaco ou acidente vascular prévio.^11,12^ Esta calculadora foi desenvolvida pelo *American College of Cardiology* (ACC) e pela *American Heart Association* (AHA), e oferece uma maneira simplificada de seguir o algoritmo de tratamento de DCVs de acordo com dados clínicos e laboratoriais e risco estratificado.^11,13-16^ As variáveis com seus respectivos intervalos consistem em idade (40-79 anos), sexo (masculino/feminino), raça (afro-americano/outro), Tc (3,4-8,3 mmol/L), HDLc (0,5-2,6 mmol/L), PAS (90-200 mmHg ) e PAD (30-140 mmHg), tratamento para HAS (sim/não), DM (sim/não) e tabagismo (sim/não). Um escore específico é atribuído ao valor/resposta de cada variável. A soma desses escores resulta no escore de risco total. Definiu-se que uma pessoa possui alto risco de um evento de DCV em dez anos quando o escore PCER calculado era ≥ 7,5%.^11^ Estimar o risco de DCV por meio da PCER não é recomendado para pessoas consideradas de risco muito alto precocemente, o que inclui pacientes com eventos de DCV conhecidos (história de síndrome coronariana aguda, infarto do miocárdio, angina estável, revascularização coronariana/outra, acidente vascular encefálico (AVE), ataque isquêmico transitório ou doença arterial periférica por aterosclerose) e pessoas com níveis extremamente elevados de LDLc (≥ 5,0 mmol/L) . Portanto, a ferramenta PCER é apropriada apenas para pessoas sem eventos de DCV prévios e com níveis de LDLc de 1,8-4,9 mmol/L.^11,12^ Finalmente, a ferramenta PCER oferece recomendações de tratamento para dislipidemia, controle da pressão arterial e prevenção de DCV segundo as diretrizes do ACC/AHA 2013.^11^

A ferramenta FRS foi desenvolvida com base no risco preditivo de DCV em um grande estudo de coorte.^11,15,17,18^ Esta ferramenta prevê o risco de dez anos de grandes eventos de DCV (doença coronariana - doença arterial crônica, acidente vascular encefálico, doença arterial obstrutiva periférica, ou insuficiência cardíaca).^11,15,17,18^ As seguintes variáveis foram inseridas em uma calculadora on-line (http://www.zunis.org/FHS_CVD_Risk_Calc_2008.htm): idade, sexo, Tc, HDLc, PAS, tabagismo e tratamentos para HAS e DM. Um escore específico é atribuído a uma característica (por exemplo, “sim” ou “não”) ou um valor para cada variável. A soma desses pontos resulta na estimativa de risco para DCV do paciente. O FRS estimado para eventos de DCV em dez anos foi categorizado como alto risco (> 20%), risco intermediário (5-20%) e baixo risco (< 5%). Conforme declarado pela ferramenta,^15^ pacientes com doença arterial coronariana, cerebrovascular ou aterosclerótica obstrutiva periférica, com manifestações subclínicas (ou seja, documentadas por metodologia diagnóstica) ou clínicas (eventos de DCV), procedimentos de revascularização arterial, DM ou doença renal crônica (taxa de filtração glomerular estimada inferior a 60 mL/min/1,73 m^2^) foram considerados de alto risco precocemente. Sendo assim, não calculamos seu risco usando a ferramenta FRS. Além disso, pessoas com risco intermediário, cuja condição foi agravada por pelo menos um fator agravante, foram reclassificadas como de alto risco.^15^ Os fatores agravantes foram (a) síndrome metabólica e (b) história familiar de DCV prematura. Por fim, os pacientes com baixo risco estimado e história familiar positiva para DCV prematura foram reclassificados para a categoria de risco intermediário.^18^

### Análise estatística

Avaliamos os dados existentes sem realizar ajustes pela falta de dados. A distribuição de normalidade foi avaliada pelo teste de Kolmogorov-Smirnov. Devido ao pequeno tamanho da população, as distribuições não foram paramétricas. Consequentemente, as variáveis contínuas foram expressas como medianas e intervalo interquartílico (IIQ). As diferenças entre os grupos foram avaliadas por um teste não paramétrico (teste U de Mann-Whitney). As variáveis categóricas foram apresentadas como frequências absolutas e relativas (porcentagens). As diferenças entre as frequências foram avaliadas pelo teste χ^2^ de Pearson. A concordância entre a ferramenta PCER e a FRS foi avaliada pelo teste do coeficiente κ de Cohen. A força de concordância foi definida de acordo com a média do coeficiente κ: ruim (< 0,00), leve (0,01-0,20), regular (0,21-0,40), moderada (0,41-0,60), substancial (0,61-0,80) e quase perfeita (0,81-1,00). Um valor de p < 0,05 foi aceito como estatisticamente significativo para todas as comparações. Os dados foram analisados por meio do software SPSS® Statistical, versão 26 (IBM, Armonk, EUA).

## Resultados

### Características do paciente

**Figura 1:**
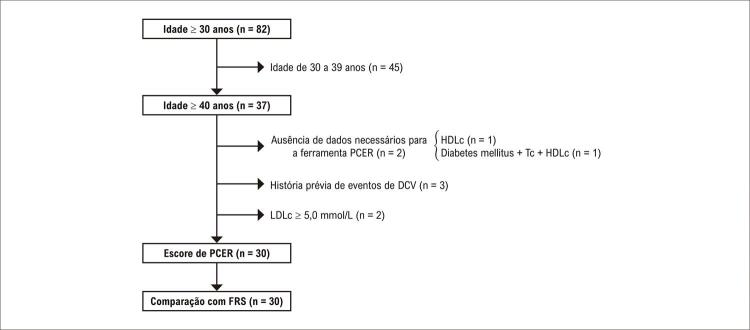
– Inclusão de pacientes de acordo com o estudo e os critérios da ferramenta PCER (Pooled Cohort Equations Risk) Calculator. A: anos; HDLc: colesterol lipídico de alta densidade; Tc: colesterol total; DCV: doença cardiovascular; LDLc: colesterol lipídico de baixa densidade; FRS: Escore de Risco de Framingham.

Trinta e sete PCHs foram incluídas (Figura 1). O valor de LDLc estava ausente para um paciente e o consideramos abaixo de 5,0 mmol/L. Dois pacientes foram excluídos da análise devido à ausência de dados de fatores de risco para DCV. Cinco das 35 PCHs restantes foram excluídas porque apresentavam risco muito alto precocemente (três tinham histórico de evento cardiovascular e dois tinham níveis extremamente altos de LDLc). Duas PCH tinham níveis de Tc abaixo da faixa adequada para a ferramenta PCER, sendo reconsiderados como o nível imputável mais baixo (3,4 mmol/L). A análise final incluiu 30 (81%) PCHs.

A idade mediana [faixa interquartil; IIQ] das PCHs foi de 51,5 [IIQ; 46,0-59,5] anos (Tabela 1). Doze (43%) PCH eram graves, 80% tinham hemofilia A, 57% estavam em profilaxia e 57% tinham infecção atual ou anterior por VHC.

**Tabela 1:** – Perfis hemofílicos e cardiovasculares e escore estimado da PCER

Características	Todos os pacientes (n = 30)	Faltosos	PCER < 7,5% (n = 15)	PCER ≥ 7,5% (n = 15)	Valor de p
**Dados clínicos**					
Idade (anos)	51,5 [46,0-59,5]	0	47,0 [45,0-51,0]	59 [54,0-62,0]	<0,001*
Branco	11/30 (37)	0	5/15 (33)	6/15 (40)	0,705^†^
Idade no momento do diagnóstico de hemofilia (anos)	18,5 [13,8-25,0]	4	15,0 [9,5-19,0]	21,0 [18,0-26,0]	0,007*
Hemofilia A	24/30 (80)	0	14/15 (93)	10/15 (67)	0,068^†^
Hemofilia grave	13/30 (43)	0	6/15 (40)	7/15 (47)	0,713^†^
Profilaxia	17/30 (57)	0	9/15 (60)	8/15 (53)	0,713^†^
Inibidor positivo	2/30 (7)	0	1/15 (7)	1/15 (7)	1,000^†^
HIV positivo	0/30 (0)	0	0/15 (0)	0/15 (0)	--
HCV positivo	17/30 (57)	0	6/15 (40)	11/15 (73)	0,065^†^
**Perfil de risco cardiovascular**					
Circunferência da cintura (cm)	91,5 [82,3-97,5]	2	88,5 [80,8-94,5]	92,0 [84,3-103,0]	0,329*
IMC (Kg/m^2^)	24,0 [22,0-27,3]	0	24,0 [22,0-26,0]	25,0 [21,0-31,0]	0,653*
Obesidade	6/30 (20)	0	2/15 (13)	4/15 (27)	0,361^†^
Tabagista	4/30 (13)	0	1/15 (7)	3/15 (20)	0,283^†^
PAS (mmHg)	134,0 [116,0-140,0]	0	120,0 [112,0-136,0]	140,0 [126,0-141,0]	0,041*
PAD (mmHg)	83,5 [80,0-90,8]	0	82,0 [76,0-93,0]	86,0 [81,0-90,0]	0,713*
Medicação anti-hipertensiva	14/30 (47)	0	4/15 (27)	10/15 (67)	0,028^†^
HAS	20/30 (67)	0	7/15 (47)	13/15 (87)	0,020^†^
Glicemia (mmol/L)	5,8 [5,1-6,5]	0	5,2 [4,9-5,8]	6,3 [5,8-11,0]	0,002*
Medicação para controle da glicose	5/29 (17)	1	0/14 (0)	5/15 (33)	0,018^†^
Diabetes mellitus	7/29 (24)	1	0/15 (0)	7/14 (50)	0,002^†^
TG (mmol/L)	1,1 [0,9-1,6]	2	1,0 [0,8-2,8]	1,1 [1,0-1,5]	0,821*
Hipertrigliceridemia	4/28 (14)	2	4/15 (27)	0/13 (0)	0,044^†^
Tc (mmol/L)	4,9 [4,1-6,0]	0	5,2 [4,3-6,0]	4,6 [3,6-6,0]	0,233*
Hipercolesterolemia	14/30 (47)	0	8/15 (53)	6/15 (40)	0,464^†^
LDLc (mmol/L)	2,8 [2,1-3,6]	1	2,8 [2,1-3,4]	2,8 [1,8-4,0]	0,847*
HDLc (mmol/L)	1,4 [1,0-1,6]	0	1,5 [1,2-1,6]	1,3 [0,9-1,5]	0,116*
HipoHDLemia	7/30 (23)	0	2/15 (13)	5/15 (33)	0,195^†^
Estatina	0/29 (0)	1	0/14 (0)	0/15 (0)	--
Síndrome metabólica	8/28 (29)	2	3/14 (21)	5/15 (36)	0,403^†^
AAS	0/30 (0)	0	0/15 (0)	0/15 (0)	--

Variáveis contínuas foram expressas como mediana [intervalo interquartílico]. As frequências foram expressas como afetados/total (%). *Teste U de Mann-Whitney; ^†^Teste do χ^2^ de Pearson. HIV: vírus da imunodeficiência humana; VHC: vírus da hepatite C; IMC: índice de massa corporal; PAS: pressão arterial sistólica; PAD: pressão arterial diastólica; HAS: hipertensão arterial sistêmica; TG: triglicerídeos; Tc: colesterol total; LDLc: colesterol lipídico de baixa densidade; HDLc: colesterol lipídico de alta densidade; AAS: ácido acetilsalicílico; PCER: Pooled Cohort Equations Risk; NA: não aplicado.

### Perfil cardiovascular

Um total de seis (20%) PCHs eram obesas e quatro (13%) eram tabagistas (Tabela 1). A HAS foi diagnosticada em 67% das PCHs, e 47% estavam em tratamento anti-hipertensivo. Sete (24%) PCHs tinham DM. A hipertrigliceridemia foi identificada em 14% das PCHs. Embora nenhuma PCH estivesse utilizando estatina, 47% apresentavam hipercolesterolemia e 23% apresentavam hipoHDLemia. Oito (29%) PCHs tinham síndrome metabólica. Nenhuma PCH estava em tratamento com ácido acetilsalicílico (AAS).

### Estimativas de risco cardiovascular

O escore mediano [IIQ] da PCER foi de 6,9 [IIQ; 3,1-13,2], e metade das PCHs apresentava alto risco de evento cardiovascular em dez anos (Tabela 1). As PCHs com alto risco de DCV segundo a ferramenta PCER eram mais velhas do que aquelas sem alto risco (p < 0,001). Além disso, tinham PAS (p = 0,041) e HAS (p = 0,020) mais altos e estavam mais frequentemente em tratamento anti-hipertensivo (p = 0,028) do que PCHs que não apresentavam alto risco de DCV segundo a ferramenta PCER. A mediana da glicemia em jejum (p = 0,002) e a prevalência de pessoas em tratamento antidiabético (p = 0,018) e com DM (p = 0,002) foram maiores entre PCHs de alto risco segundo a PCER, em comparação com suas contrapartes. Por fim, a prevalência de PCHs com hipertrigliceridemia foi menor entre PCHs com alto risco de DCV segundo a ferramenta PCER, em comparação com aqueles sem alto risco de DCV (p = 0,044).

Houve uma concordância moderada de 80% entre os instrumentos PCER e FRS [κ = 0,60 ± 0,15 (IC 95%; 0,31-0,89); p = 0,001]: Quarenta por cento (40%) foram considerados de alto risco em ambas as ferramentas e 40% foram considerados de não alto risco em ambas (Tabela 2).

**Tabela 2:** – Concordância entre as estimativas de risco cardiovascular das ferramentas *Pooled Cohort Equations Risk* e Escore de Framingham em pessoas com hemofilia com 40 anos de idade ou mais*

Ferramenta		n (%)

PCER	FRS	
**Concordância**		
alto risco	alto risco	12/30 (40%)
não alto risco	não alto risco	12/30 (40%)
	Concordância total	24/30 (80%)
**Discordância**		
alto risco	não alto risco	3/30 (10%)
não alto risco	alto risco	3/30 (10%)
	Discordância total	6/30 (20%)

Teste do coeficiente κ de Cohen, κ = 0,60 ± 0,15 (IC 95%, 0,31-0,89), valor-p (χ^2^ de Pearson) = 0,001. *Considerou-se alto risco de DCV em dez anos conforme as respectivas diretrizes: risco considerado no PCER quando ≥ 7,5%,^11^ e no FRS quando > 20,0%. Valores de risco inferiores a esses foram considerados não risco alto para eventos de DCV em dez anos.^18^ PCER: Pooled Cohort Equations Risk; FRS: Escore de Risco de Framingham; DCV: doença cardiovascular.

### Recomendações

Entre as 30 PCHs, os tratamentos com AAS e estatina foram recomendados para quatro (14%) e 16 (54%) delas, respectivamente (Tabela 3). A pressão arterial estava mal controlada em 14 (47%), das quais seis pacientes não estavam em tratamento para HAS e foram indicados para iniciá-lo. As outras oito PCHs faziam uso de anti-hipertensivos e foram orientadas a intensificar o tratamento. Três (10%) PCHs receberam indicação de AAS e estatina de alta intensidade juntamente com melhor controle da pressão arterial.

**Tabela 3:** – Recomendações da ferramenta PCER para todos os 30 pacientes, de acordo com o escore de risco estimado em dez anos*

Recomendação	n (%)	Ação necessária
**Recomendação de AAS**		
Nenhum benefício	19 (63%)	Nenhuma ação necessária
Possível benefício (considere discutir)	7 (23%)	Considere iniciar o AAS
Benefício	4 (14%)	Iniciar AAS
**Recomendação de estatina**		
Nenhuma indicação	14 (46%)	Nenhuma ação necessária
Regime de intensidade moderada/moderada a alta	8 (27%)	Iniciar estatina
Regime de alta intensidade	8 (27%)	Iniciar estatina
**Recomendação de pressão arterial**		
**Bem controlada**	16 (53%)	
sem medicamentos anti-hipertensivos	10 (63%)	Nenhuma ação necessária
com medicamentos anti-hipertensivos	6 (27%)	Nenhuma ação necessária
**Mal controlada**	14 (47%)	
sem medicamentos anti-hipertensivos	6 (43%)	Iniciar medicamentos anti-hipertensivos
com medicamentos anti-hipertensivos	8 (57%)	Ajustar medicamentos anti-hipertensivos
Benefício AAS + estatina regime de alta intensidade + pressão arterial mal controlada	3 (10%)	Iniciar AAS (todos os três) Iniciar estatina (todos os três) Iniciar medicamentos anti-hipertensivos (1) ou ajustar medicamentos anti-hipertensivos (2)

*As recomendações foram baseadas nas diretrizes do ACC/AHA.^11^ PCER: Pooled Cohort Equations Risk; AAS: ácido acetilsalicílico.

## Discussão

Mostramos que metade das PCHs assintomáticas, com 40 anos ou mais, apresentava alto risco de eventos cardiovasculares nos dez anos seguintes, de acordo com a ferramenta PCER.^11,12^ Resultados comparáveis foram obtidos quando usamos a ferramenta FRS. Além disso, metade das PCH deve realizar tratamento com estatina e/ou deve ter seu tratamento da pressão arterial otimizado, de acordo com as diretrizes do ACC/AHA.^11,13,15^ Até onde sabemos, esta é a primeira publicação em que uma ferramenta foi usada para avaliar o risco de DCV entre PCHs, adicionando recomendações internacionais de tratamento à estimativa final.

Alguns de nossos resultados corroboram estudos anteriores sobre o perfil de fatores de risco para DCV entre PCHs, embora a prevalência pareça maior para algumas características. Biere-Rafi et al.^19^ avaliaram os fatores de risco de DCV entre 100 PCHs (67% tinham 40 anos ou mais e 24% eram graves). Metade dessa população tinha HAS, mas pouquíssimos tinham dislipidemia.^19^ Uma coorte holandesa/britânica com 709 PCHs (com idades variando de 30 a 88 anos) mostrou uma prevalência de 49% de HAS, 15% de obesidade e 6% de DM.^20^

Os modelos de predição de risco de DCV foram projetados para avaliar o risco individual de um primeiro evento de DCV na população em geral. No entanto, ressalvas importantes devem ser consideradas ao usar tais escores de risco. Em primeiro lugar, o ACC/AHA desenvolveu a ferramenta PCER para estimar os riscos para o desenvolvimento de um primeiro evento de DCV em dez anos e ao longo da vida.^11,12^ Participantes de diversos grandes estudos de coorte foram finalmente incluídos para análise e desenvolvimento de equações.^11,12,21^ No entanto, pode haver uma limitação significativa quando usado em populações que não se assemelham à população de origem quanto ao interesse e às características sociais, culturais e étnicas (por exemplo, homens de Recife/Brasil).^12,21^ Em segundo lugar, como esperado devido à raridade, pessoas com distúrbios hemorrágicos hereditários não foram incluídas em nenhum dos estudos referidos, ^12,21^ o que poderia argumentar contra seu uso para predizer risco em PCHs, por exemplo. Finalmente, foi relatado que a ferramenta PCER superestimou sistematicamente os riscos em cerca de 75-150% com base em seu desempenho em cinco coortes de validação externa.^21^ Isso provavelmente se deve ao uso de dados de coortes realizadas há mais de duas décadas, que podem não refletir os níveis atuais de morbidade ou as melhorias na saúde geral e nos cuidados de saúde desde então.^21^ Isso sugere a necessidade de realizar novos estudos de validação externos para qualquer um desses modelos de avaliação de risco em coortes contemporâneas para manutenção do valor preditivo do modelo. Pennells et al.^22^ recentemente realizaram essa recalibração, mas não tivemos acesso a esse documento atualizado antes de iniciar o Estudo HemoCardio.

Essas desvantagens podem ser ilustradas pela publicação de van der Valk et al.^23^ Uma incidência de DCV menor do que a esperada, conforme avaliada pelo escore QRISK2-2011,^20-24^ foi encontrada após o acompanhamento de 579 PCHs assintomáticas com 30 anos ou mais por cinco anos (redução absoluta do risco de 2,4%).^23^ O fenótipo hemorrágico da hemofilia pode ter favorecido a menor incidência de eventos de DCVs. No entanto, o QRISK2-2011 não foi validado para PCHs, assim como as terapias para evitar eventos de DCV (por exemplo, dietas, exercícios, anti-hipertensivos e estatinas) após a avaliação do fator de risco de DCV. Avaliamos o risco de DCV usando as ferramentas PCER e FRS.^11,12,15,17,18^Acompanharemos prospectivamente esses pacientes para avaliar seus resultados.

No entanto, ferramentas de risco são amplamente utilizadas para promover uma discussão sobre mudança de comportamento e instigar o tratamento medicamentoso.^11,13,15,25^ A ferramenta PCER sugeriu terapia com AAS para 11 (37%) PCHs no Estudo HemoCardio, de acordo com as diretrizes do ACC/AHA.^11^ Até o momento, não existem ensaios clínicos randomizados sobre a segurança e a eficácia dos antitrombóticos para a prevenção primária de eventos de DCV em PCHs. A prescrição de agentes antiplaquetários ou anticoagulantes deve ser considerada por uma equipe composta por um cardiologista e um hematologista, juntamente com a administração eficaz e segura de fatores de coagulação.^8,9,26^ Um estudo francês multicêntrico, aberto e não intervencionista comparou o risco de eventos hemorrágicos em PCHs sob terapia antitrombótica (ambos agentes antiplaquetários ou anticoagulação) para prevenção secundária de DCVs para PCHs sem terapia antitrombótica (sem evento DCV prévio).^27^ O risco de hemorragias foi semelhante entre os grupos, embora hemorragia grave tenha ocorrido em ambos.^27^ No entanto, não há informação sobre reposição de fator durante os tratamentos. Sendo assim, o equilíbrio entre tratamentos anti e pró-coagulantes pode ser um desafio entre PCHs.

Enquanto quase metade das PCHs era hipercolesterolêmica e 23% eram hipoHDLêmicos, nenhum paciente estava em tratamento com estatina. A ferramenta PCER sugeriu o tratamento com estatina para 16 (54%) pacientes, oito dos quais deveriam receber um regime de alta intensidade.^11,15^ De fato, não há ensaios clínicos disponíveis sobre a segurança das estatinas para PCHs. Uma declaração recente da AHA sobre a segurança e a tolerabilidade das estatinas sugeriu que as estatinas mais eficazes poderiam produzir uma redução média no LDLc de 55% a 60% na dosagem máxima na população em geral.^28^ O risco de lesão muscular grave induzida por estatina foi menor de 0,1%, e o risco de hepatotoxicidade grave foi ainda menor.^28^ Além disso, embora o uso das estatinas como prevenção secundária possa aumentar o risco de AVE hemorrágico em pessoas com AVE isquêmico,^28^ esse não parece ser o caso para a prevenção primária.^29,30^ Assim, o tratamento com estatina em intensidades moderada ou alta parece ser uma boa estratégia para prevenir eventos cardiovasculares em PCHs.

Das 19 (63%) PCHs com HAS, 74% (14/19) tinham PA mal controlada, incluindo oito que tomavam medicamentos anti-hipertensivos. Há evidências de que sexo masculino, HAS e envelhecimento não são apenas fatores de risco para doenças aterotrombóticas,^11^ mas também fatores de risco para AVE hemorrágico.^31^ Sua associação com doença hemorrágica hereditária, principalmente quando o paciente não está em profilaxia e/ou é inibidor positivo,^32^ pode aumentar significativamente o risco de AVE hemorrágico espontâneo. Uma vez que não existem ensaios clínicos sobre a segurança e a eficácia de medicamentos anti-hipertensivos para PCHs, e esses medicamentos não estão relacionados a um risco aumentado de hemorragia, entendemos que a PCH com pressão alta não controlada deve ser monitorada de perto (por exemplo, mudanças comportamentais, medicação e verificação regular de adesão), para normalizar a PA de acordo com as diretrizes internacionais.^13^

Este estudo apresentou diversas limitações. Primeiramente, conforme discutido acima, nenhuma ferramenta populacional foi formalmente validada para prever o risco individual de DCV em PCHs. Além disso, não existem ensaios clínicos sobre a melhor opção de manejo na prevenção primária de eventos cardiovasculares entre PCHs. E, por fim, a prescrição de AAS deve ser cuidadosamente discutida com outros especialistas (por exemplo, um cardiologista) individualmente, devido ao risco de eventos hemorrágicos em PCHs. Em segundo lugar, nossos resultados referem-se a uma população específica e pequena de um único centro, o que afeta a generalização dos resultados. Atualmente, estamos planejando um estudo multicêntrico para avaliar o risco de DCV em uma população maior. Finalmente, a infecção pelo VHC pode ter influenciado os resultados, uma vez que um grande estudo recente mostrou que ela está associada a um aumento de 2,5-3,5% no risco absoluto de DCV em dez anos.^33^ No entanto, essa associação não foi confirmada por dois grandes estudos que avaliaram fatores de risco para DCV e eventos entre PCHs.^34,35^

## Conclusão

Nesta análise do Estudo HemoCardio, a prevalência de HAS e dislipidemias entre PCHs sem DCV, com 40 anos ou mais, foi significante. Portanto, metade desses pacientes apresentou um alto escore de PCER em dez anos. Hematologistas podem ser os únicos médicos em contato regular com PCHs e, portanto, nossa orientação é que sigam as diretrizes do ACC/AHA para a avaliação do risco de DCV para prevenção primária,^11^ e recomendamos que avaliem os fatores de risco de DCV tradicionais e estimem o risco de DCV em dez anos (ferramentas PCER ou FRS) a cada 4-6 anos. Sempre que um risco de DCV é diagnosticado, os hematologistas podem tratá-lo e/ou encaminhar para um especialista. Neste ambiente, os cardiologistas podem ser profissionais importantes.
