# Supplementary material for: Escores de Risco Cardiovascular entre Adultos Assintomáticos com Hemofilia
Source: Arq Bras Cardiol. 2023 Sep 4;120(9):e20230004. [Article in Portuguese] doi: 10.36660/abc.20230004 (PMC10519352; doi:10.36660/abc.20230004)
Supplement: Translation

# Translation: Cardiovascular Risk Scores among Asymptomatic Adults with Haemophilia

**Authors:** Ricardo Mesquita Camelo, Camila Caram-Deelder, Bruna Pontes Duarte, Marilia Carolina Braga de Moura, Neuza Cavalcanti de Moraes Costa, Iris Maciel Costa, Ana Maria Vanderlei, Tania Maria Rocha Guimarães, Samantha Gouw, Suely Meireles Rezende, Johanna van der Bom

**Affiliations:** 1 Brazil Faculdade de Medicina, Universidade Federal de Minas Gerais, Belo Horizonte, MG – Brazil; 2 Brazil Fundação de Hematologia e Hemoterapia de Pernambuco (HEMOPE), Recife, PE – Brazil; 3 The Netherlands Department of Clinical Epidemiology, Leiden University Medical Center, Leiden – The Netherlands; 4 The Netherlands Jon J van Rood Center for Clinical Transfusion Research, Sanquin/LUMC, Leiden – The Netherlands; 5 Brazil Faculdade de Enfermagem Nossa Senhora das Graças, Universidade de Pernambuco, Recife, PE – Brazil; 6 The Netherlands Department of Pediatric Hematology, Emma Children’s Hospital, Amsterdam UMC, University of Amsterdam, Amsterdam – The Netherlands

**Keywords:** Hemophilia A, Hemophilia B, Primary Prevention, Heart Disease Risk Factors

## Abstract

**Background:**

The mortality rate of Brazilian people with haemophilia (PwH) is decreasing, but the relative incidence of deaths associated with cardiovascular disease (CVD) is increasing.

**Objectives:**

We aimed to describe the CVD risk score of PwH according to Pooled Cohort Equations Risk (PCER) Calculator tool and its treatment recommendations. We also compared the PCER estimates with the respective Framingham Risk Score (FRS).

**Methods:**

This cross-sectional study included male PwH ≥ 40 years treated at the Comprehensive Haemophilia Treatment Centre of Pernambuco (Recife/Brazil). PwH with a previous CVD event or a low-density lipid cholesterol ≥ 5.0 mmol/L were excluded. Interviews, medical file reviews, and blood tests were performed. The PCER tool was used to estimate the CVD risk and compare it with the respective FRS. A p-value < 0.05 was accepted as statistically significant.

**Results:**

Thirty PwH were included. Median age was 51.5 [interquartile range-IQR; 46.0-59.5] years. The prevalence of obesity, systemic arterial hypertension, diabetes mellitus, hypertriglyceridaemia, hypercholesterolaemia, and hypoHDLaemia were 20%, 67%, 24%, 14%, 47%, and 23%, respectively. The median PCER score was 6.9% [IQR; 3.1-13.2], with 50% having a high risk (PCER ≥ 7.5%). Statin use was suggested for 54% of PwH. Blood pressure was poorly controlled in 47% of PwH. The agreement between PCER and FRS was 80% (κ = 0.60; p = 0.001).

**Conclusions:**

Half of the male people with haemophilia aged 40 years or older had a 10-year high risk of developing CVD with strong recommendations to improve control of dyslipidaemia and blood pressure.

**Central Illustration:** : Cardiovascular Risk Scores among Asymptomatic Adults with Haemophilia

Since the development of more effective and safe products to treat haemophilia, people with haemophilia are growing older. In addition, these individuals are developing cardiovascular diseases which are characteristic of ageing people. We propose that Haematologists and other clinicians that treat people with haemophilia formally and routinely evaluate cardiovascular risk factors among these individuals and, when present, the diseases should be treated according to estabilished protocols. When required, the patients should be referred to a specialist. PCER: pooled cohort equations risk.

## Introduction

Haemophilia is a hereditary X-linked rare bleeding disorder characterized by the reduced or absent activity of coagulation factor VIII (in haemophilia A) or factor IX (in haemophilia B).^1^ In 2020, there were 209,614 people with haemophilia (PwH) worldwide, of which 165,379 had haemophilia A, 33,076 had haemophilia B, and the remaining 11,159 had unknown haemophilia type.^2^ The most common clinical presentation of the disease is spontaneous bleeding, mainly in the joints but also in other sites (e.g., epistaxis or central nervous system).^1^ Therefore, haemophilia is considered a potentially severe disease because of its morbidities and mortality.

Factor replacement therapy provided an increase in the life expectancy of PwH.^3-5^ Therefore, PwH are living longer and the incidence of cardiovascular events (e.g., myocardial infarction and ischaemic stroke) is increasing.^3-5^ According to international guidelines, cardiovascular diseases (CVD) should be treated with antithrombotic drugs during the acute event and for secondary prevention.^6,7^ However, there are no randomized controlled trials on the optimal treatment of CVD among PwH. Physicians may base their treatments on expert opinions, balancing the risks of bleeding (due to avoiding or reducing factor replacement prophylaxis and/or prescribing anti-thrombotic medications) and clotting (due to prescribing factor replacement prophylaxis and/or avoiding anti-thrombotic medications).^8,9^ Identifying and treating CVD risk factors to prevent it may pose fewer challenges than treating a CVD event *per se* because weight control, cessation of smoking, treatments of systemic arterial hypertension (SAH), diabetes mellitus (DM), and dyslipidaemia as primary prevention are not associated with increased risk of haemorrhages.

Brazil has one of the largest populations with haemophilia worldwide (n = 13,149).^2^ In the last decade, after the adoption of the standard-of-care recommendations for haemophilia treatment, the life expectancy of Brazilian PwH has increased.^5^ In consequence, CVD-related mortality is proportionally increasing too.^5^ The aim of the current analysis of the HemoCardio Study was to describe the CVD risk score among PwH according to Pooled Cohort Equations Risk (PCER) Calculator tool and its treatment recommendations. A secondary analysis compared these results with the Framingham Risk Score (FRS).

## Method

### Study design, setting, and patient eligibility

The cross-sectional HemoCardio Study was held at the Comprehensive Haemophilia Treatment Centre of Pernambuco (CHTC-HEMOPE), in Recife/Brazil. In 2016, there were 711 PwH registered in Pernambuco state, 227 of whom were 40 years or older, and approximately 76 were followed up at the CHTC-HEMOPE.^10^ The study was offered to all men with haemophilia who were 30 years or older and registered at the outpatient clinic during their elective consultation at the CHTC-HEMOPE between August 1^st^, 2018 and July 31^st^, 2019, resulting in 82 participants. In the current analysis, data from men with haemophilia who were 40 years or older were used since this represents the target age group for the PCER evaluation (Central Illustration). Patients with a history of CVD or low-density lipid cholesterol (LDLc) of 5.0 mmol/L or higher were excluded since these characteristics indicate a very high risk of CVD event in advance, and PCER calculation is not recommended for such cases.^11,12^ All data were collected using a standardized form.

### Haemophilia-related data

A detailed description of the haemophilia-related data is found in the Supplemental Material.

### Cardiovascular risk factor profile

A detailed description of the cardiovascular risk factor profile is found in the Supplemental Material.

### Cardiovascular risk estimation tools

The PCER Calculator tool (www.cvriskcalculator.com) was used to estimate CVD 10-year risk (heart disease or stroke), assuming the person had not had a prior heart attack or stroke.^11,12^ This calculator was developed by the American College of Cardiology (ACC) and the American Heart Association (AHA), and it provides a simplified way to follow the American CVD treatment algorithm according to clinical and laboratory data and stratified risk.^11,13-16^ The variables with their respective ranges consist of age (40-79 years), gender (male/female), race (African American/other), Tc (3.4-8.3 mmol/L), HDLc (0.5-2.6 mmol/L), SBP (90-200 mmHg) and DBP (30-140 mmHg), treatment for SAH (yes/no), DM (yes/no), and smoking status (yes/no). A specific score is assigned for the value/response of each variable. The sum of these scores provides the total risk score. A person was defined as having a high risk of a 10-year CVD event when the calculated PCER score was ≥ 7.5%.^11^ Estimating CVD risk by PCER is not recommended for very high-risk people in advance, which includes patients with known CVD events (defined as a history of acute coronary syndrome, myocardial infarction, stable angina, coronary/other arterial revascularization, stroke, transient ischaemic attack, or peripheral artery disease from atherosclerosis) and people with extremely high LDLc levels (≥ 5.0 mmol/L). Therefore, the PCER tool is only appropriate for people without previous CVD events and with LDLc levels of 1.8-4.9 mmol/L.^11,12^ Finally, the PCER Calculator tool provides treatment recommendations for dyslipidaemia, blood pressure control, and CVD prevention to the 2013 ACC/AHA guideline.^11^

The FRS tool was developed based on the predictive CVD risk of a large cohort study.^11,15,17,18^ This tool predicts the 10-year risk of major CVD events (coronary disease – chronic arterial disease, stroke, peripheral obstructive arterial disease, or heart failure).^11,15,17,18^ The following variables were inputted into a web-based calculator (http://www.zunis.org/FHS_CVD_Risk_Calc_2008.htm): age, gender, Tc, HDLc, SBP, smoking status, and treatments for SAH and DM. A specific score is assigned to a characteristic (e.g., “yes” or “no”) or a value for each variable. The sum of these points provides the CVD risk estimate of the patient. The estimated FRS for 10-year CVD events was categorised into high (> 20%), intermediate (5-20%), and low risk (< 5%). As stated by the tool,^15^ patients with coronary artery, cerebrovascular, or peripheral obstructive atherosclerotic disease, with subclinical (i.e., documented by diagnostic methodology) or clinical manifestations (CVD events), arterial revascularization procedures, DM, or chronic kidney disease (estimated glomerular filtration rate lower than 60 mL/min/1.73 m^2^) were considered as high risk in advance. Hence, we did not calculate their risk using the FRS tool. Additionally, individuals with intermediate risk, whose condition was aggravated by at least one aggravating factor, were reclassified as having high risk.^15^ The aggravating factors were (a) metabolic syndrome, and (b) family history of premature CVD. Finally, patients with an estimated low risk with a positive family history of premature CVD were reclassified to the intermediate risk category.^18^

### Statistical analysis

We evaluated the existing data without performing adjustments for the missing data. Normality distribution was evaluated by the Kolmogorov–Smirnov test. Due to the small size of the population, the distributions were non-parametric. Consequently, continuous variables were expressed as medians and interquartile range (IQR). The differences between groups were evaluated by a non-parametric test (Mann-Whitney’s *U* test). Categorical variables were presented as absolute and relative (percentage) frequencies. Differences between frequencies were evaluated by Pearson’s χ^2^ test. The agreement between the PCER and FRS tool was evaluated by the Cohen’s κ coefficient test. The strength of agreement was defined according to the mean κ coefficient: poor (< 0.00), slight (0.01-0.20), fair (0.21-0.40), moderate (0.41-0.60), substantial (0.61-0.80), and almost perfect (0.81-1.00). A p-value < 0.05 was accepted as statistically significant for all comparisons. Data were analysed using SPSS® Statistical software, version 26 (IBM, Armonk, USA).

## Results

### Patient characteristics

Thirty-seven PwH were included (Figure 1). LDLc value was missing for one patient, and we considered it below 5.0 mmol/L. Two patients were excluded from the analysis due to the absence of CVD risk factor data. Five of the remaining 35 PwH were excluded because they had very high risk in advance (three had a history of CVD event, and two had extremely high levels of LDLc). Two PwH had Tc levels below the range suitable for the PCER tool, and we reconsidered them as the lowest imputable level (3.4 mmol/L). The final analysis included 30 (81%) PwH.

**Figure 1:** – Patient inclusion according to the study and PCER (Pooled Cohort Equations Risk) Calculator tool criteria. Y: years; HDLc: high-density lipid cholesterol; Tc: total cholesterol; CVD: cardiovascular disease; LDLc: low-density lipid cholesterol; FRS: Framingham Risk Score.

The median [interquartile range; IQR] age of the PwH was 51.5 [IQR; 46.0-59.5] years (Table 1). There were 12 (43%) severe PwH, 80% had haemophilia A, 57% were on prophylaxis, and 57% had current or previous HCV infection.

**Table 1:** – Haemophilia and cardiovascular profiles and PCER estimated score

Characteristic	All patients (n = 30)	Missing	PCER < 7.5% (n = 15)	PCER ≥ 7.5% (n = 15)	p-value
**Clinical data**					
Age (years)	51.5 [46.0-59.5]	0	47.0 [45.0-51.0]	59 [54.0-62.0]	<0.001*
White	11/30 (37)	0	5/15 (33)	6/15 (40)	0.705^†^
Age at diagnosis of haemophilia (years)	18.5 [13.8-25.0]	4	15.0 [9.5-19.0]	21.0 [18.0-26.0]	0.007*
Haemophilia A	24/30 (80)	0	14/15 (93)	10/15 (67)	0.068^†^
Severe haemophilia	13/30 (43)	0	6/15 (40)	7/15 (47)	0.713^†^
Prophylaxis	17/30 (57)	0	9/15 (60)	8/15 (53)	0.713^†^
Inhibitor positive	2/30 (7)	0	1/15 (7)	1/15 (7)	1.000^†^
HIV positive	0/30 (0)	0	0/15 (0)	0/15 (0)	--
HCV positive	17/30 (57)	0	6/15 (40)	11/15 (73)	0.065^†^
**Cardiovascular risk profile**					
Waist circumference (cm)	91.5 [82.3-97.5]	2	88.5 [80.8-94.5]	92.0 [84.3-103.0]	0.329*
BMI (kg/m^2^)	24.0 [22.0-27.3]	0	24.0 [22.0-26.0]	25.0 [21.0-31.0]	0.653*
Obesity	6/30 (20)	0	2/15 (13)	4/15 (27)	0.361^†^
Current smoker	4/30 (13)	0	1/15 (7)	3/15 (20)	0.283^†^
SBP (mmHg)	134.0 [116.0-140.0]	0	120.0 [112.0-136.0]	140.0 [126.0-141.0]	0.041*
DBP (mmHg)	83.5 [80.0-90.8]	0	82.0 [76.0-93.0]	86.0 [81.0-90.0]	0.713*
Antihypertensive medication	14/30 (47)	0	4/15 (27)	10/15 (67)	0.028^†^
SAH	20/30 (67)	0	7/15 (47)	13/15 (87)	0.020^†^
Glycaemia (mmol/L)	5.8 [5.1-6.5]	0	5.2 [4.9-5.8]	6.3 [5.8-11.0]	0.002*
Lowering-glucose medication	5/29 (17)	1	0/14 (0)	5/15 (33)	0.018^†^
Diabetes mellitus	7/29 (24)	1	0/15 (0)	7/14 (50)	0.002^†^
TG (mmol/L)	1.1 [0.9-1.6]	2	1.0 [0.8-2.8]	1.1 [1.0-1.5]	0.821*
Hypertriglyceridaemia	4/28 (14)	2	4/15 (27)	0/13 (0)	0.044^†^
Tc (mmol/L)	4.9 [4.1-6.0]	0	5.2 [4.3-6.0]	4.6 [3.6-6.0]	0.233*
Hypercholesterolaemia	14/30 (47)	0	8/15 (53)	6/15 (40)	0.464^†^
LDLc (mmol/L)	2.8 [2.1-3.6]	1	2.8 [2.1-3.4]	2.8 [1.8-4.0]	0.847*
HDLc (mmol/L)	1.4 [1.0-1.6]	0	1.5 [1.2-1.6]	1.3 [0.9-1.5]	0.116*
HypoHDLaemia	7/30 (23)	0	2/15 (13)	5/15 (33)	0.195^†^
Statin	0/29 (0)	1	0/14 (0)	0/15 (0)	--
Metabolic syndrome	8/28 (29)	2	3/14 (21)	5/15 (36)	0.403^†^
ASA	0/30 (0)	0	0/15 (0)	0/15 (0)	--

Continuous variables were expressed as median [interquartile range]. Frequencies were expressed as affected/total (%). *Mann-Whitney U test; ^†^Pearson’s χ^2^ test. HIV: human immunodeficiency virus; HCV: hepatitis C virus; BMI: body mass index; SBP: systolic blood pressure; DBP: diastolic blood pressure; SAH: systemic arterial hypertension; TG: triglycerides; Tc: total cholesterol; LDLc: low-density lipid cholesterol; HDLc: high-density lipid cholesterol; ASA: acetylsalicylic acid; PCER: Pooled Cohort Equations Risk; na: not applied.

### Cardiovascular profile

A total of six (20%) PwH were obese, and four (13%) were current smokers (Table 1). SAH was diagnosed in 67% of PwH, and 47% were on antihypertensive treatment. There were seven (24%) PwH with DM. Hypertriglyceridaemia was identified in 14% of PwH. Although no PwH was on a statin, 47% had hypercholesterolaemia, and 23% had hypoHDLaemia. Eight (29%) PwH had metabolic syndrome. No PwH was on acetylsalicylic acid (ASA) treatment.

### Cardiovascular risk estimates

The median [IQR] PCER score was 6.9 [IQR; 3.1-13.2], and half of the PwH were at a high 10-year risk of CVD event (Table 1). PwH and high CVD risk on the PCER tool were older than those without high risk (p < 0.001). They also had higher SBP (p = 0.041) and SAH (p = 0.020) and were more frequently on antihypertensive treatment (p = 0.028) than PwH who did not have high risk for CVD on the PCER tool. Median fasting glycaemia (p = 0.002) and both the prevalence of individuals on antidiabetic treatment (p = 0.018) and with DM (p = 0.002) were higher among high-risk PCER PwH than in their counterparts. Finally, the prevalence of PwH with hypertriclyceridaemia was lower among PwH and high risk for CVD on the PCER tool than those without high risk for CVD on the PCER tool (p = 0.044).

There was a moderate agreement of 80% between PCER and FRS tools [κ = 0.60 ± 0.15 (95% CI; 0.31-0.89); p = 0.001]: 40% were considered high-risk in both tools, and 40% were considered non-high risk in both (Table 2).

**Table 2:** – Agreement between cardiovascular risk estimates from Pooled Cohort Equations Risk and Framingham Risk Score tools in people with haemophilia who were 40 years or older*

Tool		n (%)

PCER	FRS	
**Agreement**		
high risk	high risk	12/30 (40%)
non-high risk	non-high risk	12/30 (40%)
	Total agreement	24/30 (80%)
**Disagreement**		
high risk	non-high risk	3/30 (10%)
non-high risk	high risk	3/30 (10%)
	Total disagreement	6/30 (20%)

Cohen’s κ coefficient test, κ = 0.60 ± 0.15 (95%CI, 0.31-0.89), p-value (Pearson’s χ^2^) = 0.001. *High risks of CVD in 10 years were considered as stated by the respective guidelines: risk was considered in PCER when it was ≥ 7.5%,^11^ and in FRS when it was > 20.0%. Risk values less than these were considered non-high risk for CVD events in 10 years.^18^ PCER: Pooled Cohort Equations Risk; FRS: Framingham Risk Score; CVD: cardiovascular disease.

### Recommendations

Among 30 PwH, ASA and statin treatments were recommended for four (14%) and 16 (54%), respectively (Table 3). Blood pressure was poorly controlled in 14 (47%) PwH, of whom six patients were not under SAH treatment and were recommended to start it. The other eight PwH were taking antihypertensive drugs and were recommended to intensify the treatment. Three (10%) PwH were recommended ASA and high-intensity statin together with better control of their blood pressure.

**Table 3:** – PCER tool recommendations for all 30 patients, according to the estimated 10-year risk score*

Recommendation	n (%)	Action needed
**ASA recommendation**		
No benefit	19 (63%)	No action needed
Possible benefit (consider discussing)	7 (23%)	Consider starting ASA
Benefit	4 (14%)	Start ASA
**Statin recommendation**		
No indication	14 (46%)	No action needed
Moderate/moderate-to-high intensity regimen	8 (27%)	Start statin
High-intensity regimen	8 (27%)	Start statin
**Blood pressure recommendation**		
**Well-controlled**	16 (53%)	
without antihypertensive drugs	10 (63%)	No action needed
with antihypertensive drugs	6 (27%)	No action needed
**Poorly controlled**	14 (47%)	
without antihypertensive drugs	6 (43%)	Start antihypertensive drugs
with antihypertensive drugs	8 (57%)	Adjust antihypertensive drugs
ASA benefit + high intensity regimen statin + poorly controlled blood pressure	3 (10%)	Start ASA (all 3) Start statin (all 3) Start antihypertensive drugs (1) or adjust antihypertensive drugs (2)

*Recommendations were based on the ACC/AHA guidelines.^11^ PCER: Pooled Cohort Equations Risk; ASA: acetylsalicylic acid.

## Discussion

We showed that half of asymptomatic PwH who were 40 years or older had high risk of CVD events in the following 10 years, according to the PCER Calculator tool.^11,12^ Comparable results were obtained when we used the FRS tool. More importantly, half of PwH should be on statin treatment and/or should have their blood pressure treatment optimized, according to ACC/AHA guidelines.^11,13,15^ To the best of our knowledge, this is the first publication in which an online tool was used to evaluate CVD risk among PwH, adding international treatment recommendations to the final estimate.

Some of our results corroborate previous studies on CVD risk factors profile among PwH, although the prevalence seems higher for some characteristics. Biere-Rafi et al.^19^ evaluated CVD risk factors among 100 PwH (67% were 40 years or older, and 24% were severe). Half of their population had SAH, but very few had dyslipidaemia.^19^ A Dutch/British cohort of 709 PwH (ages ranging from 30 to 88 years) showed a prevalence of 49% of SAH, 15% of obesity, and 6% of DM.^20^

Risk prediction models in CVD were designed to assess the individual risk of a first CVD event in the general population. However, important caveats must be considered when using such risk scores. Firstly, the ACC/AHA have jointly developed the PCER Calculator tool to estimate both the 10-year and lifetime risks for developing a first CVD event.^11,12^ Participants from several large cohort studies were ultimately included for analysis and equation development.^11,12,21^ However, there may be a significant limitation when used in populations that do not resemble the source population concerning its interest, social, cultural, and ethnic characteristics (e.g., men from Recife/Brazil).^12,21^ Secondly, as expected due to the rarity, people with hereditary bleeding disorders were not enrolled in any of the referred studies,^12,21^ which could argue against its use for predicting risk in PwH, for example. Finally, it has been reported that the PCER Calculator tool systematically overestimated risks by roughly 75-150% based on its performance in five external validation cohorts.^21^ This is likely due to the use of cohort data from studies conducted over two decades ago, which may not reflect current levels of morbidity or improvements in overall health and health care since then.^21^ This suggests the need for routinely performing new external validation studies for any of these risk assessment models in contemporary cohorts to maintain model predictive value. Pennells et al.^22^ have recently performed such recalibration, but we did not have access to this updated document before starting the HemoCardio Study.

These drawbacks can be illustrated by the publication of van der Valk et al.^23^ A lower-than-expected CVD incidence evaluated by the QRISK2-2011 score^20,24^ was found after following 579 asymptomatic PwH who were 30 years or older for five years (absolute risk reduction of 2.4%).^23^The bleeding phenotype of haemophilia may have favoured the lower incidence of CVD events. However, neither the QRISK2-2011 was validated for PwH nor the therapies to avoid CVD events (e.g., diets, exercise, antihypertensives, and statins) after the CVD risk factor evaluation were considered. We evaluated CVD risk using both the PCER and the FRS tools.^11,12,15,17,18^ We will prospectively follow these patients to evaluate their outcomes.

Nevertheless, risk tools are widely used to promote a discussion about behavioural change and to instigate drug treatment.^11,13,15,25^ The PCER Calculator tool suggested ASA therapy for 11 (37%) PwH in HemoCardio Study, according to ACC/AHA guidelines.^11^ There are no randomized clinical trials so far on antithrombotic’ safety and effectiveness for primary prevention of CVD events in PwH. The prescription of antiplatelet agents or anticoagulation should be considered by a team consisting of a cardiologist and a haematologist, together with the effective and safe administration of clotting factors.^8,9,26^ A multicentre, open, non-interventional French study compared the bleeding risk in PwH under antithrombotic therapy (both antiplatelet agents or anticoagulation) for CVD secondary prevention with PwH without antithrombotic therapy (no previous CVD event).^27^ The bleeding risk was similar between groups, although severe bleeding occurred in both.^27^ Nevertheless, there was no information about factor replacement during the treatments. Therefore, among PwH, balancing between anti- and pro-coagulant treatments may be challenging.

While almost half of PwH were hypercholesterolaemic and 23% were hypoHDLaemic, no patient was on statin treatment. PCER Calculator tool suggested statin treatment for 16 (54%), of whom eight should receive a high-intensity regimen.^11,15^ Indeed, there is no clinical trial on the safety of statins in PwH. A recent AHA statement on the safety and tolerability of statins posed the most effective statins could produce a mean reduction in LDLc of 55% to 60% at the maximum dosage in the general population.^28^ The risk of statin-induced serious muscle injury was lower than 0.1%, and the risk of serious hepatotoxicity was even lower.^28^ In addition, although statins as secondary prevention could increase the risk of haemorrhagic stroke in people with ischaemic stroke,^28^ this does not seem to be true for primary prevention.^29,30^ Therefore, statin treatment at moderate or high intensities seems to be a good strategy for preventing CVD events in PwH.

Of the 19 (63%) PwH who had SAH, 74% (14/19) had a poorly controlled BP, including eight PwH who were taking antihypertensive medications. There is evidence that male sex, SAH, and ageing are not only risk factors for atherothrombotic diseases,^11^ but also risk factors for haemorrhagic stroke.^31^ Their association with a hereditary bleeding disease, mainly when the patient is not on prophylaxis and/or is inhibitor positive,^32^ may significantly increase the risk of spontaneous haemorrhagic stroke. Since there are no clinical trials on antihypertensive medication’s safety and effectiveness in PwH, and these drugs are not related to an increased risk of haemorrhage, we understand that PwH with uncontrolled high BP should be closely managed (e.g., behavioural changes, medication, and regular adherence verification), to normalize BP according to international guidelines.^13^

This study has several limitations. First of all, as discussed above, no population tool has been formally validated to predict individual CVD risk in PwH. Besides, there are no clinical trials on the best management of primary prevention of CVD events among PwH. In addition, ASA prescription should be cautiously discussed with other specialists (e.g., a cardiologist) on an individual basis due to the risk of bleeding events in PwH. Secondly, our results refer to a specific and small population from one single centre, which impacts the generalizability of the findings. We are currently planning a multicentre study to evaluate CVD risk in a larger population. Finally, HCV infection could have influenced the results since a recent large study showed that it is associated with a 2.5-3.5% increase in the 10-year CVD absolute risk.^33^ However, this association was not confirmed by two large studies evaluating CVD risk factors and events among PwH.^34,35^

## Conclusion

In this analysis of the HemoCardio Study, the prevalence of SAH and dyslipidaemia among PwH who were 40 years or older and free from CVD was not negligible. Therefore, half of these patients had a high 10-year PCER score. Since haematologists may be the only physicians to visit PwH regularly, we support them in following the ACC/AHA guidelines for the assessment of CVD risk for primary prevention,^11^ and we recommend them to assess traditional CVD risk factors and estimate 10-year CVD risk (PCER or FRS tools) every 4-6 years. Whenever a CVD risk is diagnosed, they can treat it and/or refer to a specialist. In such an environment, cardiologists may be significant professionals.
